# Navigating the Complex Intersection of Substance Use and Psychiatric Disorders: A Comprehensive Review

**DOI:** 10.3390/jcm13040999

**Published:** 2024-02-09

**Authors:** Anees Bahji

**Affiliations:** Departments of Psychiatry and Community Health Sciences & Hotchkiss Brain Institute, Cumming School of Medicine, University of Calgary, Calgary, AB T2R 1N4, Canada; anees.bahji1@ucalgary.ca

**Keywords:** comorbidity, substance use disorders, psychiatric disorders, treatment interventions, dual diagnosis

## Abstract

The co-occurrence of substance use disorders (SUDs) and psychiatric conditions, often referred to as comorbidity or concurrent disorders, presents intricate challenges in both diagnosis and treatment. This comprehensive narrative review aims to synthesize and critically evaluate the existing evidence surrounding the management of individuals with comorbid SUDs and psychiatric disorders. Comorbidity in these domains carries profound implications for clinical practice, research, and policymaking, emphasizing the need for a holistic understanding of the intricate dynamics that arise when these conditions coexist. This review explores recent research findings, evidence-based guidelines, and emerging trends within the field, offering valuable insights for clinicians, researchers, and policymakers seeking to navigate the complex terrain of comorbidity in substance use and psychiatric disorders.

## 1. Introduction

The co-occurrence of substance use disorders (SUDs) and psychiatric disorders, commonly referred to as comorbid conditions, concurrent disorders, or dual diagnoses, presents not only a significant diagnostic challenge but also substantial difficulties in selecting and providing evidence-based treatments [1]. To that end, this narrative review endeavors to offer a comprehensive synthesis and review of existing evidence pertaining to the treatment of individuals with comorbid SUDs and psychiatric conditions. Comorbidity in these domains carries substantial implications for clinical practice, research, and policymaking [2,3]. Comorbidity significantly transforms the landscape of clinical practice, demanding a comprehensive grasp of the intricate dynamics that arise when these conditions coexist. In the realm of research, comorbidity offers unique opportunities for exploration and interdisciplinary collaboration. It stimulates the scientific community to delve into the underlying mechanisms, risk factors, and treatment outcomes associated with the convergence of these disorders. It encourages the utilization of multidisciplinary approaches to enrich our comprehension of the biological, psychological, and social underpinnings.

Within this intricate landscape, healthcare providers and researchers grapple with the complicated interplay between substance misuse and psychiatric conditions. These challenges transcend the sum of their components, necessitating a nuanced and holistic approach to care. To effectively address these challenges, this review endeavors to consolidate the current state of knowledge concerning interventions. It encompasses a diverse array of approaches, including pharmacological, psychotherapeutic, and integrated strategies, with the overarching aim of providing profound insights and guidance to those at the forefront of addiction and psychiatry [4].

This review aims to delve into recent research findings, evidence-based guidelines, and emerging trends within the field. By synthesizing this wealth of information, this review seeks to construct a valuable resource for clinicians, researchers, and policymakers, assisting them in navigating the intricate terrain of comorbidity in substance use and psychiatric disorders. Through this review, we aspire to illuminate effective strategies that can enhance the well-being and outcomes of individuals confronting dual diagnoses.

## 2. Prevalence and Characteristics of Dual Diagnoses

Comorbidity, where individuals experience both psychiatric disorders and SUDs, is a common and prevalent phenomenon in the field of mental health. It is often considered the rule rather than the exception. Numerous reviews and meta-analyses have consistently demonstrated the high prevalence of comorbidity compared to the general population. For instance, approximately one in five individuals with an eating disorder will develop an SUD at some point in their lifetime, with one in ten currently meeting SUD criteria [5]. Likewise, evidence suggests a disproportionate impact of opioid use disorder (OUD) among individuals with schizophrenia, who are less likely to receive opioid agonist therapy (OAT) and tend to have a poorer prognosis [6]. Additionally, comorbidity with cannabis use disorder (CUD) is notably higher among individuals with bipolar disorder [7]. While the specific implications of comorbidity are unclear, it often creates barriers to treatment.

In a recent US study [8], using data from the National Epidemiologic Survey on Alcohol and Related Conditions Wave III, researchers compared adults with dual diagnoses to those with either psychiatric disorders or substance use disorders (SUDs). The findings showed that adults with dual diagnoses accounted for 26% of those with psychiatric disorders, 37% of those with SUDs, and 18% of the total 76 million adults with either condition. For individuals with psychiatric disorders, dual diagnosis was associated with significant social or psychopathological challenges, including violence, reduced mental health-related quality of life (HRQOL), law enforcement encounters, homelessness, and incarceration. Similarly, among those with SUDs, dual diagnosis was linked to social or psychopathological disadvantages, such as diminished mental HRQOL, childhood trauma exposure, childhood sexual abuse, specific drug use diagnoses, suicide attempts, medical issues, multiple SUD diagnoses, childhood neglect, recurring adult traumas, and reduced social support.

In a related systematic review, Tomáš and Lenka explored the epidemiology of dual diagnoses among children and adolescents primarily undergoing treatment for psychiatric conditions [9]. They found that the prevalence of dual diagnoses within this specific target population ranged from 18% to 54%, with an average prevalence of 33%. Notably, the review highlighted that males were more likely to experience dual diagnoses, and affective disorders emerged as the most prevalent psychiatric diagnoses among this group.

## 3. The Complex Nature of Comorbidity

The co-occurrence of SUDs and psychiatric conditions is characterized by the intricate interplay between the psychological, biological, and social underpinnings of these dual conditions [10]. From a diagnostic standpoint, the co-occurrence of SUDs and psychiatric conditions introduces distinct challenges, particularly when arriving at a diagnosis involving two or more comorbid disorders. For instance, stimulant use disorders, such as those involving methamphetamine, can manifest symptoms of psychosis, including auditory and visual hallucinations, paranoid delusions, disorganized thinking, and disorganized behavior. Interestingly, individuals with schizophrenia and related psychotic disorders, especially when left untreated, can present with strikingly similar symptoms. What further complicates the diagnostic process is the high prevalence of methamphetamine use among individuals with schizophrenia, leading to the perplexing ‘chicken-or-egg’ dilemma.

During such diagnostic evaluations, psychiatrists and other clinicians face the complex task of disentangling these relationships. They must determine a timeline in the history of the presenting illness while taking into account contributing factors, such as the individual’s premorbid level of functioning, family history, and substance use patterns. Often, this process poses a formidable challenge that cannot be definitively resolved during the initial assessment. Instead, it necessitates multiple evaluations over time to arrive at an accurate diagnosis.

Compounding the complexity, individuals grappling with these dual diagnoses often turn to substances for various reasons. These motivations may include attempting to alleviate symptoms of their underlying psychiatric illness, managing the side effects of their prescribed medications, or coping with the stigma associated with these disorders. Consequently, this creates a web of intricate medical, social, and psychological needs that demand comprehensive and patient-centered care [11].

To effectively address this comorbidity, a comprehensive approach extending beyond pharmacological interventions is imperative. These encompass housing, financial support, social assistance, medical care, and psychiatric care, aiming to provide a holistic framework that enhances the quality of life for individuals with dual diagnoses. It is essential to recognize that the complexity of comorbidity necessitates a multi-dimensional response and a seamless integration of multiple disciplines and services [12]. In their study, Iudici et al. explored some of the clinical intricacies of dual diagnosis, where SUDs coincide with psychological or psychiatric disorders, revealing significant issues across various thematic areas and emphasizing the need for innovative approaches and alternative theoretical frameworks in future research [13]. However, there exists no integrated framework that facilitates effective communication about dual diagnosis cases across disciplinary or sectoral boundaries. Larsen et al. (2022) explored the potential of Enactive Psychiatry (EP) to bridge this theoretical gap [10]. EP represents a specific instantiation of broader psychiatric paradigms, proposing that mental disorders are shaped by four primary categories of factors: biological, experiential, socio-cultural, and existential. In comparison to the traditional biopsychosocial model (BPSM), EP offers a more effective means of integrating these factors due to its unique approach.

## 4. The Role of Integrated Care

Effectively addressing comorbid SUDs and psychiatric conditions demands a comprehensive treatment approach [12,14]. Among the various treatment strategies available, current evidence strongly supports an approach known as integrated care. Integrated care, also referred to as parallel or comprehensive care, entails the simultaneous provision of treatment for all co-occurring disorders an individual may experience. For instance, when dealing with individuals who have comorbid alcohol use disorders and mood or anxiety disorders, both conditions are addressed concurrently.

In contrast, a less favored approach, termed serial care, dictates that an individual must first attain stability in one condition before the other can be addressed. Using the same example, an individual may be required to achieve complete abstinence from alcohol and undergo detoxification services before addressing the ‘underlying’ depression or anxiety disorder with psychotherapy, antidepressant medication, or a combination thereof.

However, the serial care model has its limitations. First, it fails to acknowledge how underlying factors can contribute to the development of both conditions, assuming a simplistic cause-and-effect relationship (e.g., suggesting that an individual’s alcohol use ‘caused’ their depression or anxiety). Second, it does not consider that during certain forms of substance detoxification, the withdrawal phase can exacerbate concurrent psychiatric disorder symptoms (e.g., individuals may experience heightened anxiety or depression during acute alcohol withdrawal, potentially leading to a resumption of alcohol use). This underscores the idea that substance use often serves a role as a form of self-medication, with individuals using substances for specific reasons and rationales.

Despite these challenges, the serial care approach still offers valuable insights that can significantly inform our understanding and treatment of concurrent disorders. One illuminating concept in this approach, often likened to a “Trojan Horse”, has been articulated by experts such as Dr. Anna Lembke, a prominent psychiatrist directing the Stanford University Concurrent Disorders Program. The term “Trojan Horse” in this context draws an analogy to the legendary tale of the Trojan War. In this ancient story, the Greeks used a massive wooden horse as a deceptive strategy to gain entry into the city of Troy. Similarly, in the field of concurrent disorders, some patients enthusiastically engage with concurrent disorder programs with a similar notion. They believe that by focusing on and addressing their underlying psychiatric comorbidities, like depression or anxiety, they are essentially introducing a ‘hidden’ solution that will surreptitiously infiltrate and resolve their substance use issues.

Rich Roll aptly described this analogy in his podcast interview with Dr. Anna Lembke, which can be found on YouTube [15]. In the analogy, the wooden horse represents the psychiatric comorbidity, while the city of Troy symbolizes the challenges posed by SUDs. The patients, akin to the Greeks, believe that once the ‘horse’ of psychiatric treatment is inside, it will reveal its true potential to address their substance use concerns.

However, what makes this analogy particularly insightful is the realization that much like the original Trojan Horse, the situation can be more complex. As the ‘horse’ (psychiatric treatment) enters, it may indeed address and alleviate the psychiatric symptoms, but it does not necessarily guarantee the complete resolution of substance use issues. This unfolding scenario emphasizes the need for a comprehensive and nuanced approach to treating concurrent disorders, acknowledging that these conditions may coexist independently rather than one being solely responsible for the other.

In specific scenarios, Dr. Lembke and her team may cautiously consider this approach. For instance, if a patient reports underlying depression or anxiety, they might initiate antidepressant treatment and closely monitor the patient’s progress over time. However, what is particularly insightful is how this approach unfolds when the client’s depression or anxiety begins to improve, as indicated by standardized rating scales and clinical evaluations, yet their alcohol use persists.

This divergence between the amelioration of psychiatric symptoms and the continued engagement in alcohol use provides valuable insights for both the patient and the clinicians. It suggests that alcohol use may represent a separate, concurrent issue rather than being exclusively attributable to an underlying psychiatric illness. This nuanced perspective reevaluates SUDs as distinct illnesses that do not always have a direct causal link to another psychiatric disorder. Instead, it underscores their concurrent nature, recognizing that these disorders can coexist independently, and one may not necessarily be a direct consequence of the other.

In essence, the “Trojan Horse” concept highlights the importance of individualized treatment approaches that acknowledge the multifaceted nature of concurrent disorders. It emphasizes the need to address each condition comprehensively while remaining open to the possibility that the relationship between them may be more complex than initially assumed. This approach not only guides clinical practice but also contributes to a more nuanced understanding of the intricate interplay between substance use and psychiatric conditions.

However, integrated care transcends the realms of medication and psychotherapy, encompassing a broader spectrum of care domains that address clients’ individual biopsychosocial needs. It acknowledges that effective treatment should extend beyond traditional therapeutic modalities. For instance, in cases where clients face significant social inequities, the inclusion of interventions such as access to stable housing, financial support, and peer support becomes equally as vital and impactful as medications or interactions with a psychiatrist. Integrated care recognizes that these external factors play a pivotal role in an individual’s overall well-being and treatment outcome [16]. To that end, the treatment of individuals with comorbid SUDs and psychiatric disorders necessitates a comprehensive and integrated care approach [17]. Integrated care models, which combine SUD and mental health treatment, have demonstrated efficacy in improving outcomes for this complex population [18]. These models acknowledge that individuals with dual diagnoses often face overlapping challenges that cannot be effectively addressed in isolation [19]. Studies have shown that individuals receiving integrated care experience enhanced treatment engagement, reduced substance use, improved mental health, and an overall better quality of life [20,21].

## 5. Pharmacological Interventions

Pharmacological interventions play a pivotal role in the treatment of SUDs and psychiatric conditions [22]. Medications designed to address both substance misuse and mental health symptoms can be integral components of individualized treatment plans [23]. While some individuals may benefit from medications targeting substance cravings or withdrawal symptoms, others may require psychiatric medications to manage mood and anxiety disorders [24,25,26,27]. The careful selection and monitoring of medications, along with ongoing evaluation of their effectiveness, are crucial aspects of treatment [28]. Pharmacological interventions, including medications such as clozapine, have shown promise in treating individuals with comorbid SUDs and psychiatric conditions, with research suggesting higher odds of abstinence and reduced psychiatric hospitalization; however, the effectiveness of such treatments remains inconclusive due to limited sample sizes and insufficient reporting in randomized controlled trials, emphasizing the importance of integrating medication into a comprehensive treatment approach that includes psychotherapeutic interventions and social support [29,30,31]. By leveraging the potential benefits of pharmacological interventions within a broader treatment framework, we can optimize outcomes for individuals with dual diagnoses [32,33].

## 6. Psychotherapeutic Approaches

Medication alone seldom serves as a comprehensive solution for addressing comorbidity, underscoring the importance of integrating it into a broader treatment framework that encompasses psychotherapeutic interventions and social support [34,35,36]. Psychosocial interventions have demonstrated significant promise in the treatment of individuals with comorbid substance use and psychiatric disorders [37,38]. Several meta-reviews evaluating the efficacy of psychotherapies for SUDs, spanning a range of substances such as alcohol, cannabis, stimulants, opioids, and benzodiazepines, have consistently shown that psychosocial treatments yield small to moderate benefits compared to inactive controls in the short-term [39,40,41,42,43,44].

Evidence-based strategies, including contingency management (CM), cognitive-behavioral therapy (CBT), dialectical behavior therapy (DBT), motivational interviewing (MI), acceptance and commitment therapy (ACT), and family-based interventions, have demonstrated their effectiveness in reducing substance use and enhancing treatment outcomes by addressing the psychological, behavioral, and social aspects of addiction [45,46,47,48,49,50,51,52,53,54,55,56]. For instance, CBT focuses on helping individuals modify maladaptive thought patterns and behaviors associated with both substance use and psychiatric symptoms. At the same time, DBT equips patients with crucial skills for emotional regulation and distress tolerance, which are vital in navigating the complexities of comorbidity.

For example, a systematic review by Lee et al. aimed to identify effective treatment options for individuals with comorbid substance use and borderline personality disorders, with findings suggesting that both DBT and dynamic deconstructive psychotherapy (DDP) exhibited some beneficial effects in symptom reduction [46]. MI fosters intrinsic motivation for change and aids individuals in exploring their ambivalence towards substance use and mental health management. The effectiveness of 12-step group facilitation interventions, such as Alcoholics Anonymous (AA), in addressing SUDs, particularly alcohol use disorder (AUD), is supported by high-quality evidence [57], showing their efficacy in promoting abstinence. However, their effectiveness in addressing comorbid SUDs remains uncertain. Nevertheless, additional research in this domain is imperative. For instance, Cochrane reviews have consistently highlighted the insufficiency of evidence to establish the effectiveness of certain psychotherapies for various SUDs, whether accompanied by comorbidity or not. An example of this shortfall is the inadequacy of high-quality research regarding the effectiveness of CBT in the treatment of stimulant use disorders [47,48,58].

## 7. Harm Reduction Strategies

In the realm of comorbid SUDs and psychiatric conditions, harm reduction strategies emerge as a pragmatic and compassionate approach to care. This dual-diagnosis population faces unique challenges, often influenced by social determinants of health, pervasive stigma, and limited access to comprehensive care. In this context, harm reduction strategies offer a holistic approach that prioritizes safety and well-being over immediate abstinence, recognizing that complete sobriety may not always be immediately achievable or realistic.

Harm reduction interventions encompass a range of evidence-based practices, including supervised consumption sites, naloxone distribution programs, and proactive outreach initiatives. These interventions aim to reduce the negative consequences associated with substance use while simultaneously addressing the complex mental health needs of individuals facing comorbidity. By creating non-judgmental and supportive environments, harm reduction strategies foster trust and rapport, enabling individuals to engage with healthcare services at their own pace and make gradual improvements in their lives.

It is important to note that while harm reduction has made substantial strides in addressing the challenges faced by individuals with SUDs, its impact on those with comorbid conditions remains less well-defined. Therefore, further research is necessary to assess the effectiveness of harm reduction interventions specifically tailored to individuals grappling with both SUDs and psychiatric disorders. Embracing harm reduction as a fundamental principle of care ensures that we meet individuals where they are on their journey toward recovery, offering a compassionate and effective pathway that recognizes the complexities of dual diagnosis scenarios.

## 8. Addressing Comorbidity in Alcohol Use Disorders and Anxiety/Depression

As previously discussed, there exists a substantial overlap between alcohol use disorders (AUDs) and mood/anxiety disorders, a common presentation encountered by psychiatrists. Yet, the approach to treating individuals with these concurrent disorders is intricate, primarily due to several challenging factors. One significant challenge arises from the difficulty in accurately identifying specific subgroups within this population. Distinguishing individuals with concurrent disorders from those with alcohol-induced mood/anxiety disorders, those experiencing mood/anxiety symptoms as a result of alcohol withdrawal, or those with one disorder but not necessarily the other, can be a complex endeavor.

The literature has yielded mixed findings regarding the effectiveness of particular pharmacological treatments, especially Selective Serotonin Reuptake Inhibitors (SSRIs). This complexity is further underscored by the recent Canadian guideline on high-risk drinking and AUD management, which raises concerns regarding the use of SSRIs in individuals with AUD and non-substance-induced Major Depressive Disorder (MDD) or anxiety disorders [59]. While the guideline references specific studies to support its recommendations, it is essential to recognize that these studies may not fully capture the potential benefits of SSRIs for this particular population.

In fact, some research suggests that combining SSRIs with AUD treatment can yield significant benefits, emphasizing the critical importance of addressing both conditions concurrently [60,61,62]. Moreover, Cochrane reviews have highlighted the potential utility of SSRIs in treating MDD, anxiety disorders, AUD, or combinations of these conditions in individuals with co-occurring AUD, with minimal risk of adverse effects [63,64]. Left untreated, MDD among individuals with AUD can have profound consequences [65].

Hence, rather than imposing a blanket restriction on the use of SSRIs for individuals with dual disorders, a judicious and case-by-case evaluation is recommended. In complex cases where diagnostic ambiguity exists, referring the individual to a psychiatrist with expertise in addiction can be particularly beneficial. This specialized assessment can help untangle intricate diagnostic scenarios and guide treatment decisions effectively.

However, it is crucial to acknowledge that, for many individuals, SSRIs are a valuable treatment option. They can be a lifeline for those grappling with depression or anxiety. It is important to recognize that untreated depression and anxiety are significant risk factors for alcohol use disorder. Moreover, it is essential to differentiate between the common co-occurrence of these conditions and the notion that SSRIs directly ‘cause’ alcohol use.

In essence, the approach should be individualized, considering the unique circumstances and needs of each person. This approach not only recognizes the complex interplay between these disorders but also emphasizes the importance of tailored interventions that promote the overall well-being of individuals with dual diagnoses.

## 9. Addressing Comorbidity in Stimulant Use Disorders and ADHD

While concerns have arisen regarding the over-diagnosis of many psychiatric conditions, diagnosing ADHD in adults without a pre-existing childhood or developmentally-oriented diagnosis is a controversial area that has received increasing attention in recent years. The nature of ADHD as a diagnostic construct contributes to these issues. For example, when an adult seeks diagnostic evaluation, clinicians must determine if the symptoms of inattention, hyperactivity, and/or impulsivity had a developmental onset (e.g., before the age of 12). This task is prone to various forms of bias, especially in the absence of collateral information or observation. The recent CADDRA guidelines recommend against relying solely on the mental status examination to support or refute an ADHD diagnosis, potentially limiting the diagnosis to self-report. However, this approach raises concerns, as it may open the door to individuals seeking secondary gain, given that the gold-standard treatment for moderate-to-severe ADHD involves the prescription of psychostimulant medication.

Further complicating matters is the tendency of some clinicians to utilize treatment response as a soft sign for diagnostic confirmation. For instance, if an individual newly diagnosed with ADHD experiences a positive response to ADHD medication, that response is sometimes used to validate the diagnosis. However, this practice lacks an evidence-based foundation, as healthy volunteers (those without ADHD) also report improved concentration and focus when taking stimulants. Stimulants promote attention and concentration in individuals both with and without ADHD, making this response an unreliable confirmation of the diagnosis.

Another concern relates to noting a patient’s therapeutic response to recreational drugs. Some clinicians believe that when patients report a calming or focusing effect from using illicit stimulants, such as methamphetamines or cocaine, instead of experiencing euphoria, it suggests that they have ADHD. However, this hypothesis also lacks an evidence base and may be influenced by bias, especially in cases where patients seek stimulants for secondary gain. This situation likely occurs to some extent and may go undetected due to lax diagnostic validation policies in many clinics, such as the absence of collateral information, limited use of the mental status examination to support clinical diagnostic evaluation, lack of urine drug screening to rule out medication diversion, and complete reliance on patient self-reporting to guide treatment.

Retroactively diagnosing ADHD in adults who can provide a reliable estimate of their childhood symptoms before the age of 12 presents a significant challenge. The nature of ADHD and the inherent fallibility of memory introduce various heuristics and potential biases. Furthermore, this approach often lacks crucial contextual information as adults may recall their adolescent selves through the lens of seeking an ADHD diagnosis, potentially missing the broader context necessary for understanding their issues. In contrast, diagnosing ADHD in childhood benefits from additional information sources, such as parental and teacher input.

While some adults legitimately receive ADHD diagnoses due to longstanding struggles and well-documented historical evidence supporting the diagnosis, many clients seek quick diagnoses without adequate validation. This trend raises concerns about the evolving threshold for psychiatric disorders over time. While it may be influenced by changing stigmas surrounding ADHD, the increasing prevalence of adults seeking these diagnoses despite lacking childhood evidence suggests the potential over-medicalization of normal experiences.

ADHD serves as a pertinent example of this dilemma. Unlike some other disorders, ADHD lacks an objective biomarker for definitive diagnosis and primarily relies on self-reported symptoms. While diagnosing children with ADHD allows for potential observation and assessment, concerns arise when neurologically normal adults receive ADHD diagnoses, often in the context of workplace challenges, such as difficulties sustaining focus on screens for extended periods. Such situations may not necessarily indicate ADHD but could represent the upper limit of normal human cognition and focus.

This situation is concerning because seeking an ADHD diagnosis solely to obtain prescription stimulants for enhanced productivity at work blurs the line between legitimate clinical need and potentially inappropriate medication use. Psychiatrists face the challenging task of distinguishing valid diagnoses from invalid ones in this controversial area, further complicated by the lack of clear-cut criteria and objective measures.

The complexity deepens when comorbid stimulant use disorder is introduced into the equation. This comorbidity can drive an ADHD diagnosis, as it provides a socially acceptable source for obtaining prescription medications. However, it can also emerge as a by-product of exposure to an addictive medication, such as a prescribed psychostimulant. The widespread belief in the dopamine agonist model for stimulant use disorder, akin to the use of opioid agonist therapies for opioid use disorder, further complicates the intricate interplay between these conditions. However, unlike the decades of evidence supporting various forms of opioid agonist therapy for treating opioid use disorder, the current body of evidence supporting prescription stimulants for treating stimulant use disorders remains inconclusive [58,66,67,68,69]. It raises doubts about their safety and efficacy, as the two forms of addiction may not necessarily share the same biological underpinnings.

Moreover, the co-occurrence of ADHD in individuals with stimulant use disorders introduces additional layers of complexity in diagnosis and treatment. This is because the symptoms of stimulant intoxication and withdrawal can closely mimic those of ADHD [70]. As a result, clinicians often approach the prescription of psychostimulants with caution, concerned about the potential for worsening substance use outcomes and the risk of misuse [69,71]. In such cases, non-stimulant medications like atomoxetine are frequently preferred despite the potential advantages offered by long-acting prescription stimulants [72,73].

While initial studies suggest that higher doses of specific stimulants may be beneficial for a subset of individuals with comorbid ADHD and stimulant use disorder, it is crucial to emphasize that more research is needed to establish the safety and efficacy of such treatments in this complex population [74]. In essence, addressing the comorbidity of stimulant use disorders and ADHD calls for a nuanced approach that carefully considers the intricate interplay of various factors and acknowledges the existing gaps in evidence [75].

## 10. Discussion and Conclusions

The coexistence of SUDs and psychiatric conditions challenges clinicians, researchers, and policymakers to adopt multifaceted approaches that acknowledge the complexity of these dual diagnoses. This review has shed light on the intricate interplay between psychological, biological, and social factors that define comorbidity. It underscores the importance of individualized care, embracing integrated treatment models, and recognizing the limitations of pharmacological interventions in isolation. Furthermore, the “Trojan Horse” concept has highlighted the need for nuanced approaches that consider the multifaceted nature of concurrent disorders.

As we move forward, it is imperative to continue exploring the effectiveness of various treatment strategies, including psychotherapeutic interventions and harm reduction strategies, in addressing comorbidity. Special attention should be given to addressing diagnostic challenges and potential over-medicalization, especially in cases of adult ADHD diagnosis. Additionally, the intersection of stimulant use disorders and ADHD requires ongoing research to determine safe and effective treatment approaches.

In conclusion, the path toward improving the well-being and outcomes of individuals confronting dual diagnoses involves embracing complexity, individualization, and holistic care. By doing so, we can provide better support and hope for those navigating the intricate landscape of comorbidity in substance use and psychiatric disorders.

## Data Availability

Data sharing is not applicable to this review as no new data were generated, and the availability of existing data is restricted by privacy and ethical considerations.
